# Supplementary material for: Preclinical Study of Plasmodium Immunotherapy Combined with Radiotherapy for Solid Tumors
Source: Cells. 2022 Nov 14;11(22):3600. doi: 10.3390/cells11223600 (PMC9688403; doi:10.3390/cells11223600)
Supplement: Supplementary file 1 [file cells-11-03600-s001.zip › cells-1942845-supplementary.pdf]

cells

MDPI

Supplementary Material

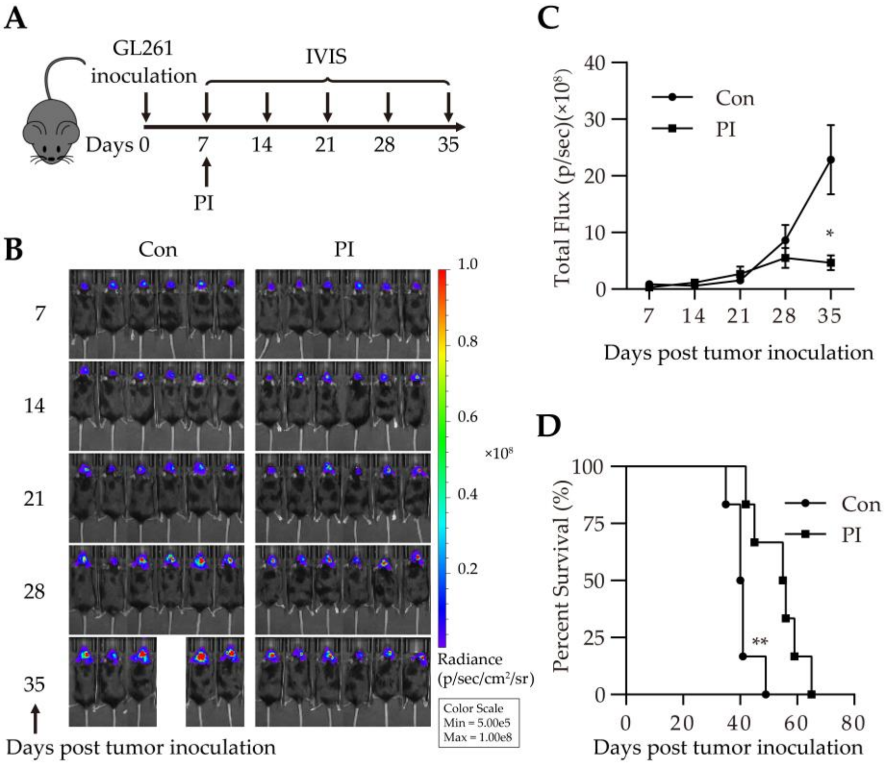

Figure S1. PI inhibited tumor growth and prolonged the survival of intracerebral GL261-Luc glioma-bearing mice. (**A**) Schematic representation of PI for the treatment of intracerebral GL261-Luc glioma in syngeneic C57BL/6J mice. (**B**) Living images and (**C**) tumor growth was assessed by total bioluminescence signals on days 7, 14, 21, 28 and 35 post inoculation of the GL261-Luc cells ($n = 6$). The data showed mean ± SEM. The statistical difference between groups at the experiment endpoint was analyzed with an unpaired two-tailed Student's t-test. (**D**) Data showed the Kaplan-Meier survival curves of mice ($n = 6$). Survival curves were analyzed by a log-rank test. Statistical differences were indicated by the p values, *, $p \leq 0.05$; **, $p \leq 0.01$; ***, $p \leq 0.001$.

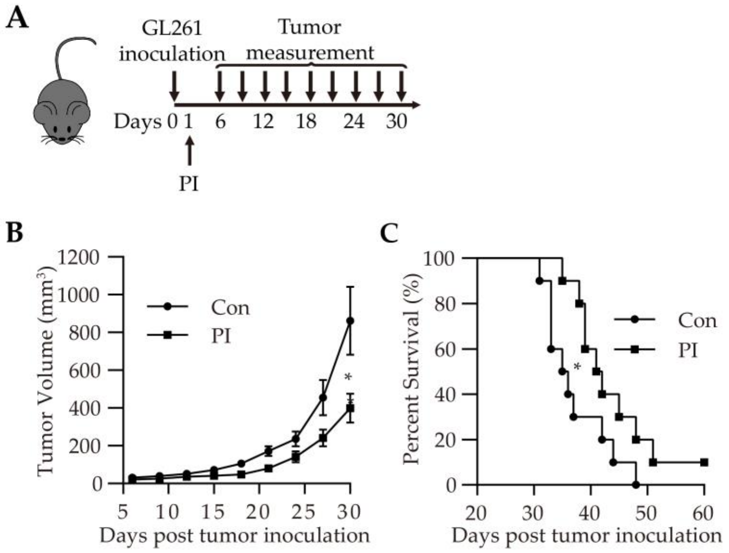

Figure S2. PI significantly inhibited tumor growth and prolonged the survival of subcutaneous GL261 glioma-bearing mice. **(A)** Schematic representation of PI for the treatment of subcutaneous GL261 glioma in syngeneic C57BL/6J mice. (**B**) Tumor growth was measured over time ($n = 10$). The data showed mean ± SEM. The statistical difference between groups in the experiment endpoint was analyzed with unpaired an unpaired two-tailed Student's t-test. (**C**) Data showed the Kaplan-Meier survival curves of mice ($n = 10$). Survival curve was analyzed by a log-rank test. Statistical differences were indicated by the p values, *, $p \le 0.05$; **, $p \le 0.01$; ***, $p \le 0.001$.

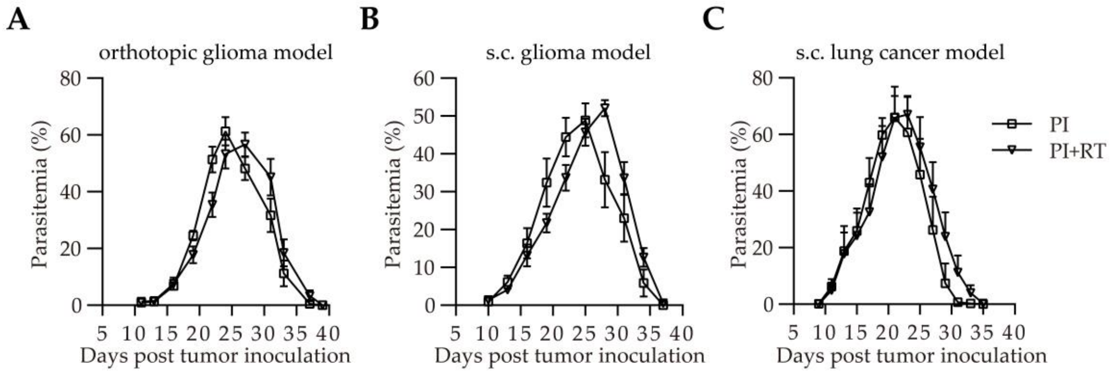

Figure S3. Dynamics of parasitemia in the mice of the combination therapy experiments. Parasitemia was determined in PI group and PI+RT group infected with *Plasmodium yoelii* 17XNL. Dynamics of parasitemia in **(A)** orthotopic GL261-Luc glioma model as shown in Figure 1, (**B**) subcutaneous GL261 glioma model as shown in Figure 2, (**C**) subcutaneous LLC lung cancer model as shown in Figure 3.

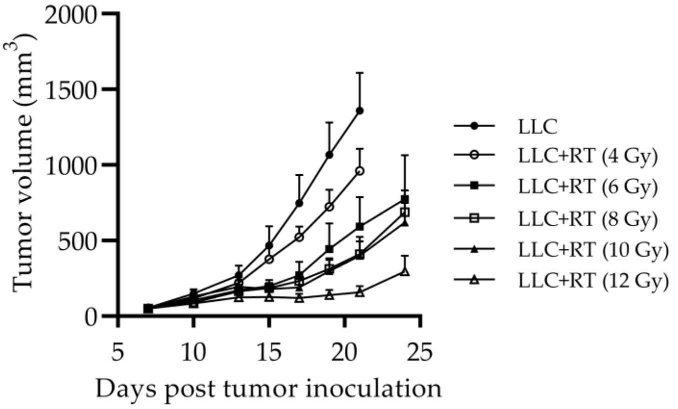

Figure S4. Effect of different X-ray dose on LLC tumor growth. Each mouse was subcutaneously implanted with 5×10^5 LLC cells. Mice received 4, 6, 8, 10 and 12 Gy of X-ray radiation treatment respectively on day 7 post tumor inoculation. Mice without receiving treatment were used as control group. Each group had 4 mice.

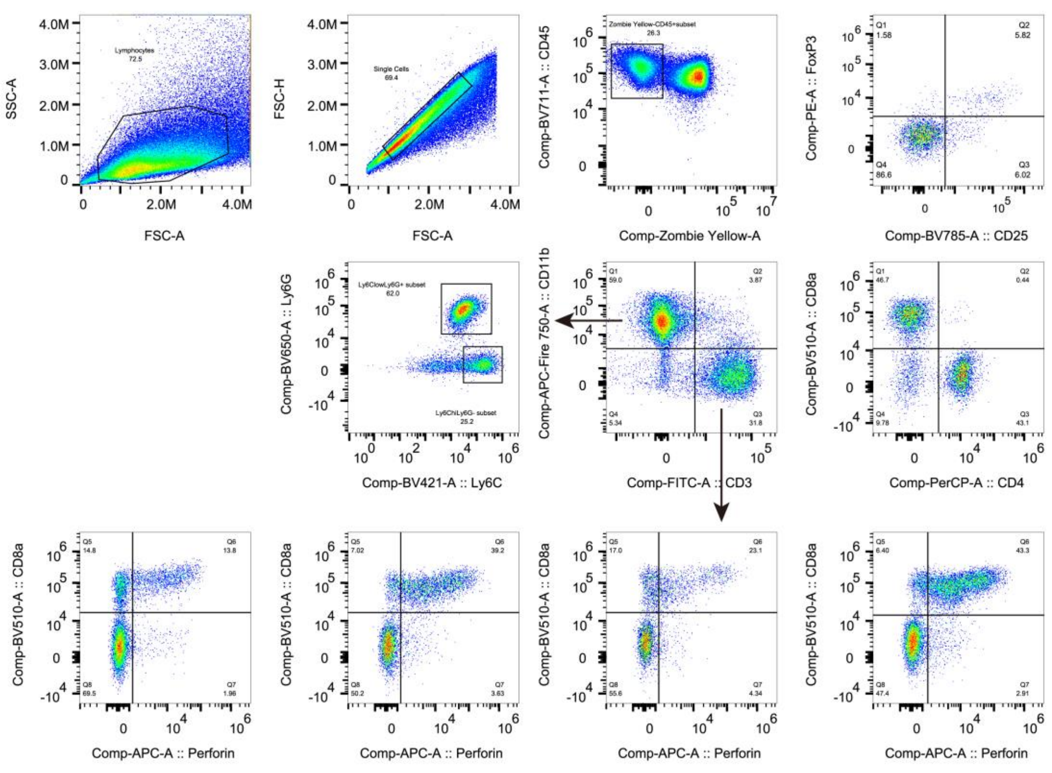

Figure S5. Representative plots of gating strategies in flow cytometry analysis.

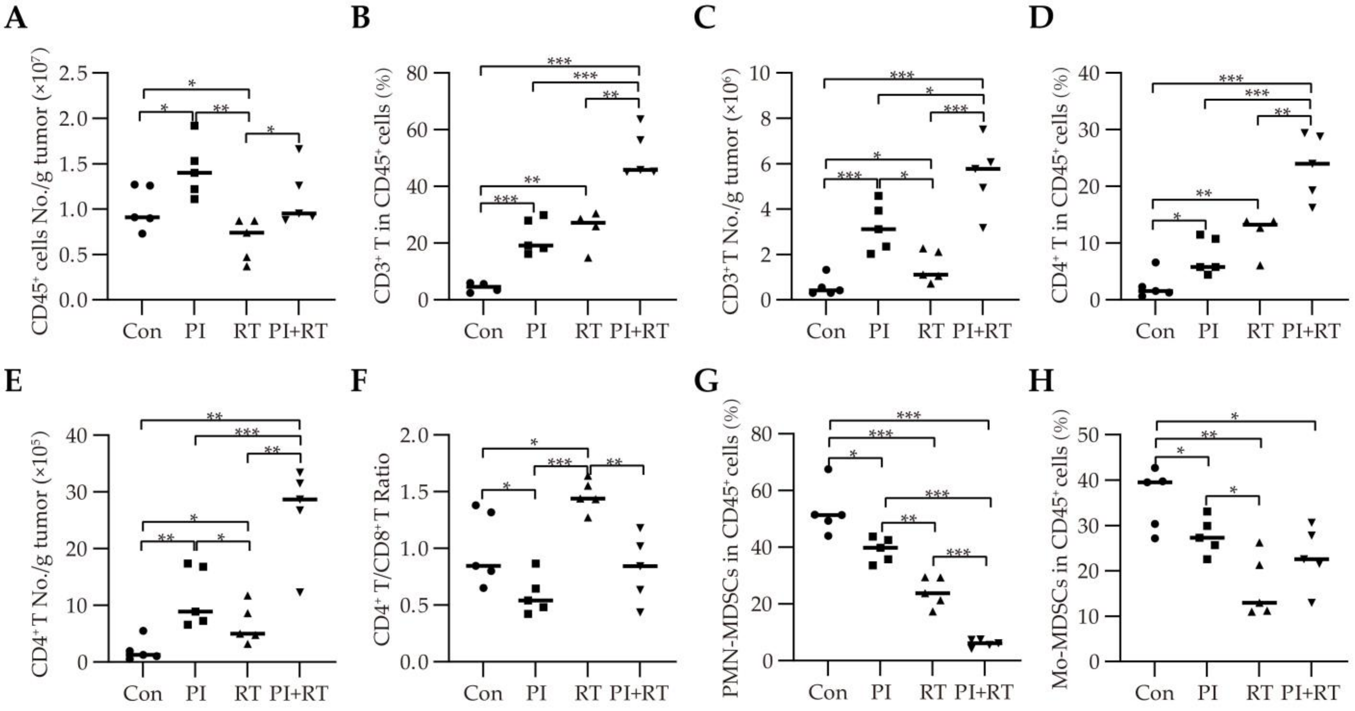

Figure S6. The effect of PI in combination with RT on the immune profiles in tumor tissue in subcutaneous LLC lung cancer-bearing mice. Lymphocytes were isolated from tumor on day19 post inoculation. (n = 5). **(A)** Absolute number of $CD45^+$ cells. (**B**) Proportion of $CD3^+$ T cells in $CD45^+$ cells. (**C**) Absolute number of $CD3^+$ T cells. (**D**) Proportion of $CD4^+$ T cells in $CD45^+$ cells. (**E**) Absolute number of $CD4^+$ T cells. (**F**) Ratio of $CD4^+$ T cells to $CD8^+$ T cells. (**G**) Proportion of PMN-MDSCs in $CD45^+$ cells. (**H**) Proportion of Mo-MDSCs in $CD45^+$ cells. Absolute number was presented as the number of cells per gram of tumor. The statistical differences between groups were analyzed with an unpaired two-tailed Student's t-test. The data showed mean ± SEM. Statistical differences were indicated by the p values, *, $p \leq 0.05$; **, $p \leq 0.01$; ***, $p \leq 0.001$.

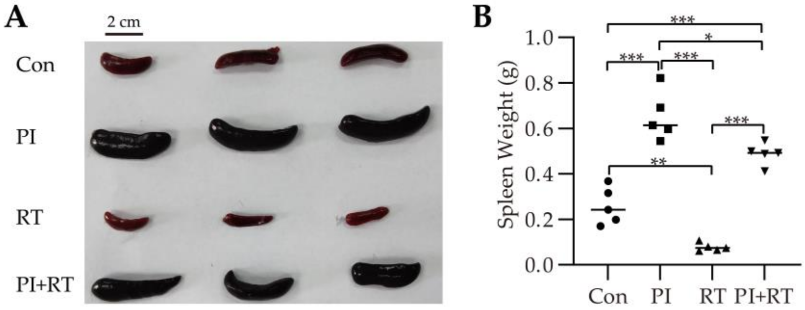

Figure S7. The effect of PI in combination with RT on the spleen weight in subcutaneous LLC lung cancer-bearing mice. The spleens were harvested on day 19 post implantation. (**A**) Comparisons of visual observation of the representative spleen sizes (n = 3). (**B**) Comparisons of the spleen weights (n = 5). The statistical differences between groups were analyzed with an unpaired two-tailed Student's t-test. The data showed mean ± SEM. Statistical differences were indicated by the p values, *, $p \leq 0.05$; **, $p \leq 0.01$; ***, $p \leq 0.001$.

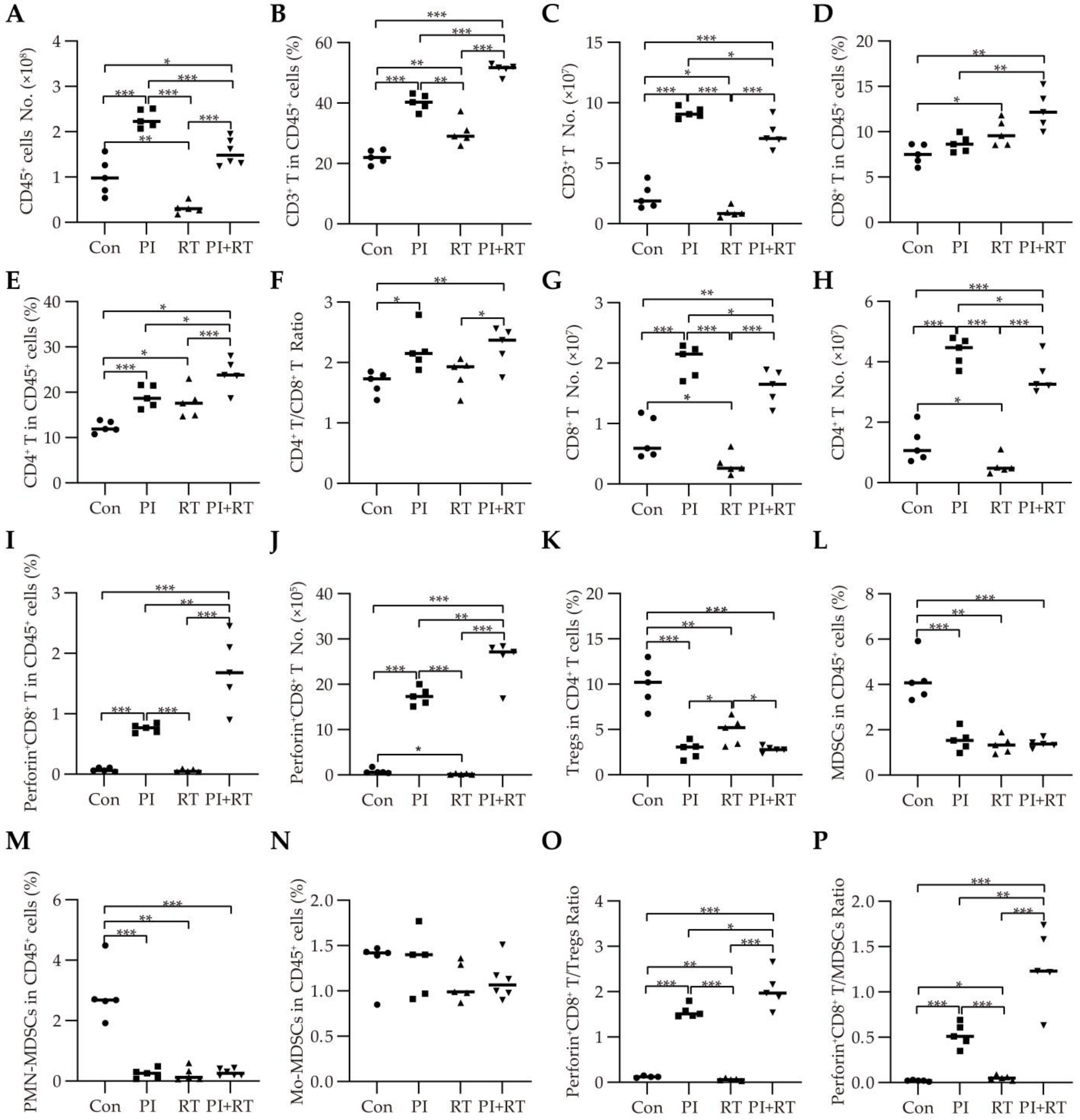

Figure S8. The effect of PI in combination with RT on the profiles in the spleens in subcutaneous LLC lung cancer-bearing mice. Lymphocytes were isolated from the spleen on day 19 post inoculation (n = 5). (**A**) Absolute number of CD45⁺ cells. (**B**) Proportion of CD3⁺ T cells in CD45⁺ cells. (**C**) Absolute number of CD3⁺ T cells. (**D**) Proportion of CD8⁺ T cells in CD45⁺ cells. (**E**) Proportion of CD4⁺ T cells in CD45⁺ cells. (**F**) Ratio of CD4⁺ T cells to CD8⁺ T cells. (**G**) Absolute number of CD8⁺ T cells. (**H**) Absolute number of CD4⁺ T cells. (**I**) Proportion of perforin⁺CD8⁺ T cells in CD45⁺ cells. (**J**) Absolute number of perforin⁺CD8⁺ T cells. (**K**) Proportion of Tregs in CD4⁺ T cells. (**L**) Proportion of MDSCs in CD45⁺ cells. (**M**) Proportion of PMN-MDSCs in CD45⁺ cells. (**N**) Proportion of Mo-MDSCs in CD45⁺ cells. (**O**) Ratio of perforin⁺CD8⁺ T cells to Tregs. (**P**) Ration of perforin⁺CD8⁺ T cells to MDSCs. The statistical differences between groups were analyzed with an unpaired two-tailed Student's t-test. Absolute number was presented as the number of cells per spleen. The data showed

mean ± SEM. Statistical differences were indicated by the *p* values, *, $p \leq 0.05$; **, $p \leq 0.01$; ***, $p \leq 0.001$.

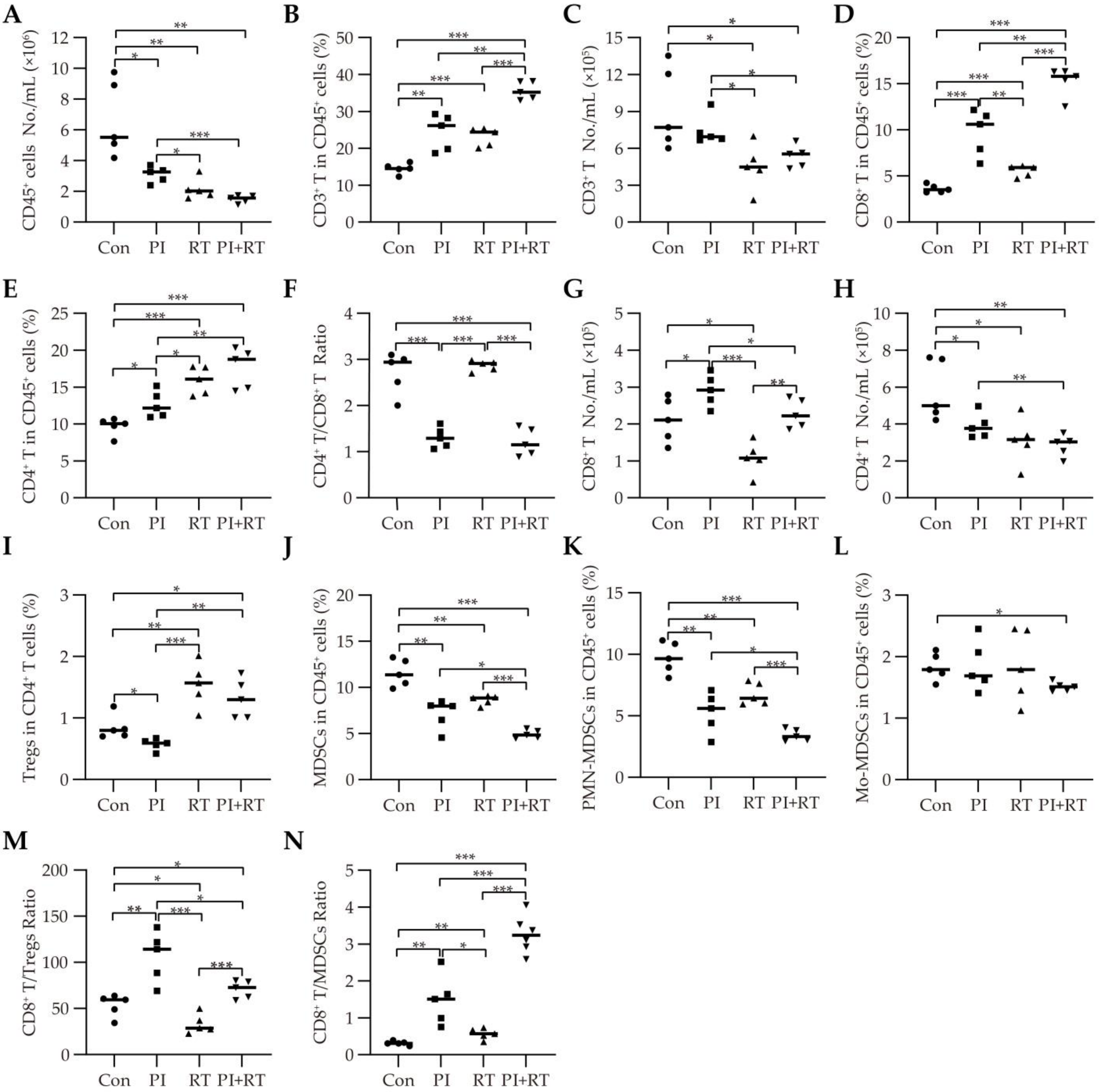

Figure S9. The effect of PI in combination with RT on the immune profiles in peripheral blood in subcutaneous LLC lung cancer-bearing mice. Lymphocytes were isolated from blood on day 19 post inoculation ($n = 5$). (**A**) Absolute number of $CD45^+$ cells. (**B**) Proportion of $CD3^+$ T cells in $CD45^+$ cells. (**C**) Absolute number of $CD3^+$ T cells. (**D**) Proportion of $CD8^+$ T cells in $CD45^+$ cells. (**E**) Proportion of $CD4^+$ T cells in $CD45^+$ cells. (**F**) Ratio of $CD4^+$ T cells to $CD8^+$ T cells. (**G**) Absolute number of $CD8^+$ T cells. (**H**) Absolute number of $CD4^+$ T cells. (**I**) Proportion of Tregs in $CD4^+$ T cells. (**J**) Proportion of MDSCs in $CD45^+$ cells, (**K**) Proportion of PMN-MDSCs in $CD45^+$ cells. (**L**) Proportion of Mo-MDSCs in $CD45^+$ cells. (**M**) Ratio of $CD8^+$ T cells to Tregs. (**N**) Ratio of $CD8^+$ T cells to MDSCs. Absolute number was presented as the number of cells per mL blood. The statistical differences between groups were analyzed with an unpaired two-tailed Student's t-test. The data showed mean ± SEM. Statistical differences were indicated by the p values, *, $p \leq 0.05$; **, $p \leq 0.01$; ***, $p \leq 0.001$.
